# Association between low values of mean arterial pressure and impaired cognitive performance in older patients with mild cognitive impairment: cross-sectional preliminary findings from the STRENGTH Project

**DOI:** 10.1007/s40520-023-02668-5

**Published:** 2024-01-28

**Authors:** Elpidio Santillo, Marta Balietti, Paolo Fabbietti, Maria Sole Antolini, Cristina Paoloni, Francesco Piacenza, Cinzia Giuli

**Affiliations:** 1 Geriatric-Rehabilitative Department, IRCCS INRCA, Fermo, Italy; 2 Center for Neurobiology of Aging, IRCCS INRCA, Via Birarelli 8, 60121 Ancona, Italy; 3 Centre for Biostatistic and Applied Geriatric Clinical Epidemiology, IRCCS INRCA, Ancona, Italy; 4 Geriatric Operative Unit, IRCCS INRCA, Fermo, Italy; 5 Advanced Technology Center for Aging Research, IRCCS INRCA, Ancona, Italy

**Keywords:** Mild cognitive impairment, Mean arterial pressure, Older adults, Cognitive performance, Neuropsychological assessment

## Abstract

**Background:**

Uncontrolled blood pressure (BP) is a risk factor for Mild Cognitive Impairment (MCI) and dementia.

**Aims:**

This study examined the relationship between BP and clinical/cognitive/neuropsychological aspects in MCI individuals.

**Methods:**

MCI patients underwent clinical, functional, cognitive and metacognitive, as well as psychological assessments. Social network, lifestyle characteristics, and medication prescriptions were also evaluated. Each patient underwent BP measurements.

**Results:**

Lower values of systolic BP (SBP), diastolic BP (DBP), and mean arterial pressure (MAP) were associated with poorer cognitive performance. Notably, MAP showed greater capability in detecting impairments in attention and visuospatial abilities compared to SBP and DBP.

**Discussion:**

These findings support the notion that in older individuals with MCI excessively low BP values, particularly MAP, might represent a risk and suggest that cerebral hypoperfusion may play a key role.

**Conclusions:**

Routine assessment of MAP could aid clinicians in adjusting antihypertensive treatment and closely monitoring cognitive function in MCI patients.

**Supplementary Information:**

The online version contains supplementary material available at 10.1007/s40520-023-02668-5.

## Introduction

Mild Cognitive Impairment (MCI) affects around 10–15% of individuals aged 65 years and older worldwide [1], with MCI subjects having a higher incidence of dementia compared to the general population [2].

One of the potential factors contributing to the progression from MCI to dementia is midlife hypertension, which has been found to increase the risk by 1.19 to 1.55 fold [3]. Although the impact of arterial hypertension on brain morphology and function, including hypertrophic vascular remodelling and cerebral hypoperfusion, is well-established [4], the association between BP and cognitive decline in older ages is not yet fully understood, partially due to its non-linear trajectory throughout life. Evidence suggests that lower BP values in older individuals are associated with increased cognitive impairment risk [5], and approximately five years prior to dementia onset, BP tends to decline [6]. Moreover, cognitively impaired older adults show a U-shaped curve between diastolic BP values and the greatest relative risk of mortality [7].

An additional challenge lies in determining the optimal BP parameter for identifying MCI patients at elevated risk, with mean arterial pressure (MAP) being the most underrated candidate. Indeed, although MAP holds promising potential being an index that defines the cerebral perfusion status by reflecting the systolic and diastolic BP during the cardiac cycle [8], its investigation in elderly individuals with MCI has been limited thus far. Furthermore, the Mini-Mental State Examination (MMSE) is usually applied to evaluate the cognitive status in MCI subjects, notwithstanding other tests, such as the Montreal Cognitive Assessment (MoCA), have demonstrated superior diagnostic capabilities [9]. Indeed, the MMSE does not always detect the alterations in executive functions, which are often early impaired in hypertensive patients [10]. Moreover, in older patients, specific tests for assessing executive function, selected and divided attention, visuospatial skills and speed of processing, such as the Trail Making Test (TMT) parts A and B, have been demonstrated to be particularly useful in capturing the progression of cognitive decline from MCI to dementia [11].

The present study examined the possible relationships between BP parameters, including MAP, and several cognitive and psychological aspects in MCI patients by applying a robust cognitive, neuropsychological and clinical assessment. The objective is to provide novel insights into the appropriate management of BP in elderly individuals who may require tailored therapeutic interventions.

## Materials and methods

Community-dwelling older adults with MCI were enrolled at the Geriatric Operative Unit of IRCCS INRCA in Fermo (Italy). The study protocol was approved by the local ethics committee (IRCCS INRCA Bioethics Advisory Committee, Ancona, Italy; code no. 18006), registered on ClinicalTrials.gov (code no. NCT04146818), and described in detail in Giuli and colleagues [12]. Briefly, the inclusion criteria comprise diagnosis of MCI [13], age 60 years or older, presence of a caregiver, and capability to sign the informed consent; the exclusion criteria include diagnosis of dementia and presence of psychiatric conditions. Every participant underwent a comprehensive face-to-face assessment, during which they provided information about their daily habits and medical history, covering previous and current main pathologies, hospitalizations, medications, and surgeries. Moreover, structured neuropsychological tools and scales were administered by appropriately trained healthcare experts. All evaluations were conducted within a single session, with each participant’s visit lasting approximately 1.5 h. In details, functional (i.e., basic activities of daily living, ADL; instrumental activities of daily living, IADL; Short Physical Performance Battery, SPPB), cognitive and metacognitive (i.e., Memory Complaint Questionnaire, MAC-Q; MoCA; MMSE; immediate and delayed Rey auditory verbal learning test, RAVLT; phonemic verbal fluency test, PVF; Corsi Supra-Span test; semantic verbal fluency test, SVF; attentive matrices; TMT A; TMT B; TMT A-B; clock drawing test), and psychological (i.e., Depression Anxiety Stress Scale, DASS; Geriatric Depression Scale-15, GDS-15; 36 health survey, SF-36) assessments were performed. Social network (i.e., Lubben Social Network Scale, LSNS), lifestyle characteristics (i.e., Physical Activity Scale for the Elderly, PASE; Body Mass Index, BMI; smoking habits; alcohol consumption), and medication prescriptions were also evaluated. Cognitive tests were adjusted for age and schooling.

Furthermore, each patient underwent three BP measurements at 2 min intervals on the right arm by manual auscultatory method, according to guidelines recommendations [14]. All BP readings were conducted during the morning (between 9 and 10 AM) within a tranquil, air-conditioned room maintained at a temperature of 22 °C to 24 °C. During the 12 h preceding the BP measurements, participants were prohibited from consuming caffeine, alcohol, or engaging in smoking. Furthermore, all participants were instructed to adhere to their regular medication routines, including any antihypertensive drugs, at their prescribed times. The average of the second and third BP readings was used for the analysis to reduce the risk of overestimation of BP values for eventual alerting reaction. Phase I and Phase V Korotkoff sounds were used to identify systolic BP (SBP) and diastolic BP (DBP), respectively. Pulse pressure (PP) was calculated as the difference between SBP and DBP, whereas MAP was calculated as DBP plus one-third of the PP. A level of SBP ≥ 140 mmHg and/or a level of DBP ≥ 90 mmHg were considered thresholds for the new diagnosis of hypertension [14].

The cohort analysed in the present study comprises a subset of individuals (n = 101) who were enrolled prior to the onset of the SARS-CoV-2 pandemic, consisting entirely of older adults who have not received vaccination and, presumably, have not been infected with the virus at the time of assessment due to their asymptomatic status and residence in a minimally affected area during the initial wave. Thus, they would serve as a reference group for investigating the potential influence of the disease and the impact of associated social restrictions on similar studies.

### Statistical analyses

The normality of variables was assessed using the Kolmogorov–Smirnov test. Data for continuous variables were reported as mean ± standard deviation for normally distributed variables and as median [interquartile range] for non-normally distributed variables. Categorical variables were presented as percentages. Pearson’s r and Spearman’s rho coefficients were used to correlate BP values and cognitive/neuropsychological parameters. Binary logistic regression was applied to evaluate the possible influence of lifestyle, drugs, and comorbidities on the relationship between BP values and cognitive/psychosocial domains. Comparisons among subgroups were performed by one-way ANOVA. Statistical significance was set at *p* < 0.05. All statistical analyses were performed with SPSS version 24 (SPSS Inc., Chicago, IL, USA).

To determine the statistical power of our study, a post hoc calculation was conducted, specifically considering the influence of MAP values on performance in TMT A test (main outcome). By utilizing the difference between two independent means (two-tailed), an effect size of 0.52 (representing the difference of MAP values between patients with a TMT A score ≤ or > the median level), and an α-error of 0.05, we obtained a power value of 0.83. By using the median to binarize the TMT A value instead of clinical scores, numerical imbalances between the subgroups were avoided, ensuring the validity of the analysis.

## Results

Table 1 summarizes the main characteristics of the MCI cohort.

**Table 1:** Socio-demographic data, clinical and functional assessment, cognitive/metacognitive and psychological profile, lifestyle, and pharmacological treatment of the MCI cohort

	Total
	(n = 101)
Age (years), median [IR]	77.0 [72.0–81.0]
Gender (% of women)	73.3
Marital status (%)	
Single	2.0
Married	48.5
Divorced/separated	10.9
Widowed	38.6
Education (years), median [IR]	8.0 [5.0–13.0]
Cardiac arrhythmia (%)	17.8
Hypertension (%)	66.3
Type 2 diabetes (%)	5.9
Osteoarthritis/rheumatoid arthritis (%)	74.3
Osteoporosis (%)	27.7
Gastritis (%)	23.8
Prostatic hypertrophy (%)	14.9
Anxiety (%)	47.5
Depression (%)	30.7
Hypothyroidism (%)	13.9
Dyslipidemia (%)	22.8
ADL, median [IR]	6.0 [6.0–6.0]
IADL, median [IR]	8.0 [8.0–8.0]
SPPB, median [IR]	9.0 [8.0–10.0]
MAC-Q, median [IR]	26.0 [25.0–28.0]
MoCA, median [IR]	22.0 [21.0–24.0]
MMSE, mean ± sd	27.3 ± 1.8
Immediate RAVLT, mean ± sd	38.6 ± 9.5
Delayed RAVTLT, median [IR]	7.1 [5.4–8.8]
PVF, mean ± sd	4.7 ± 8.3
Corsi Supra-span, median [IR]	4.5 [4.2–5.0]
SVF, median [IR]	17.7 [14.7–19.7]
Attentive matrices, mean ± sd	48.7 ± 7.4
TMT A, mean ± SD	0.7 ± 1.6
TMT B, mean ± SD	0.4 ± 1.4
TMT B-A, mean ± SD	0.3 ± 1.8
Clock drawing test, median [IR]	9.0 [7.0–9.0]
DASS depression, median [IR]	4.0 [0.0–8.0]
DASS anxiety, median [IR]	2.0 [0.0–6.0]
DASS stress, median [IR]	8.0 [4.0–12.0]
GDS-15, median [IR]	3.0 [2.0–5.0]
SF36, median [IR]	591.2 [461.1–670.4]
LSNS, mean ± SD	28.0 ± 5.5
Smoker (%)	11.1
Alchool consumption (number of daily drink), median [IR]	1.0 [0.0–2.0]
BMI (kg/m^2^), mean ± SD	25.7 ± 3.6
PASE, median [IR]	394.0 [362.8–422.9]
Benzodiazepines (% of users)	14.9
Antidepressants (% of users)	3.0
Lipid-lowering drugs (% of users)	17.8
Beta-blockers (% of users)	20.8
RAS-acting agents (% of users)*	41.6
non-RAS-acting agents (% of users)^a^	21.8
Diuretics (% of users)^b^	21.4
MAP (mmHg), mean ± SD	96.6 ± 10.8
SBP (mmHg), median [IR]	140.0 [130.0–150.0]
DBP (mmHg), mean ± sd	74.3 ± 10.4
PP (mmHg), median [IR]	63.5 [55.0–76.2]

*ADL* Basic activities of daily living, *IADL* instrumental activities of daily living, *SPPB* short physical performance battery, *MAC-Q* memory complaint questionnaire, *MoCA* montreal cognitive assessment, *MMSE* mini mental state examination, *RAVLT* rey auditory verbal learning test, *PVF* phonemic verbal fluency test, *SVF* semantic verbal fluency test, *TMT A* trail making test A, *TMT B* trail making test B, *TMT B-A* trail making test B-A, *DASS* depression anxiety stress scale, *GDS-15* geriatric depression scale-15, *SF-36* 36 health survey, *LSNS* lubben social network scale, *BMI* body mass index, *PASE* physical activity scale for the elderly, *MAP* mean arterial pressure, *SBP* systolic blood pressure, *DBP* diastolic blood pressure, *PP* pulse pressure, *RAS*: renin-angiotensin system.

*Angiotensin-converting enzyme inhibitors angiotensin II receptors antagonists

^a^Calcium channel blockers alpha 2-adrenoceptors antagonists

^b^Loop diuretics thiazides potassium-sparing diuretics

**Fig. 1:**
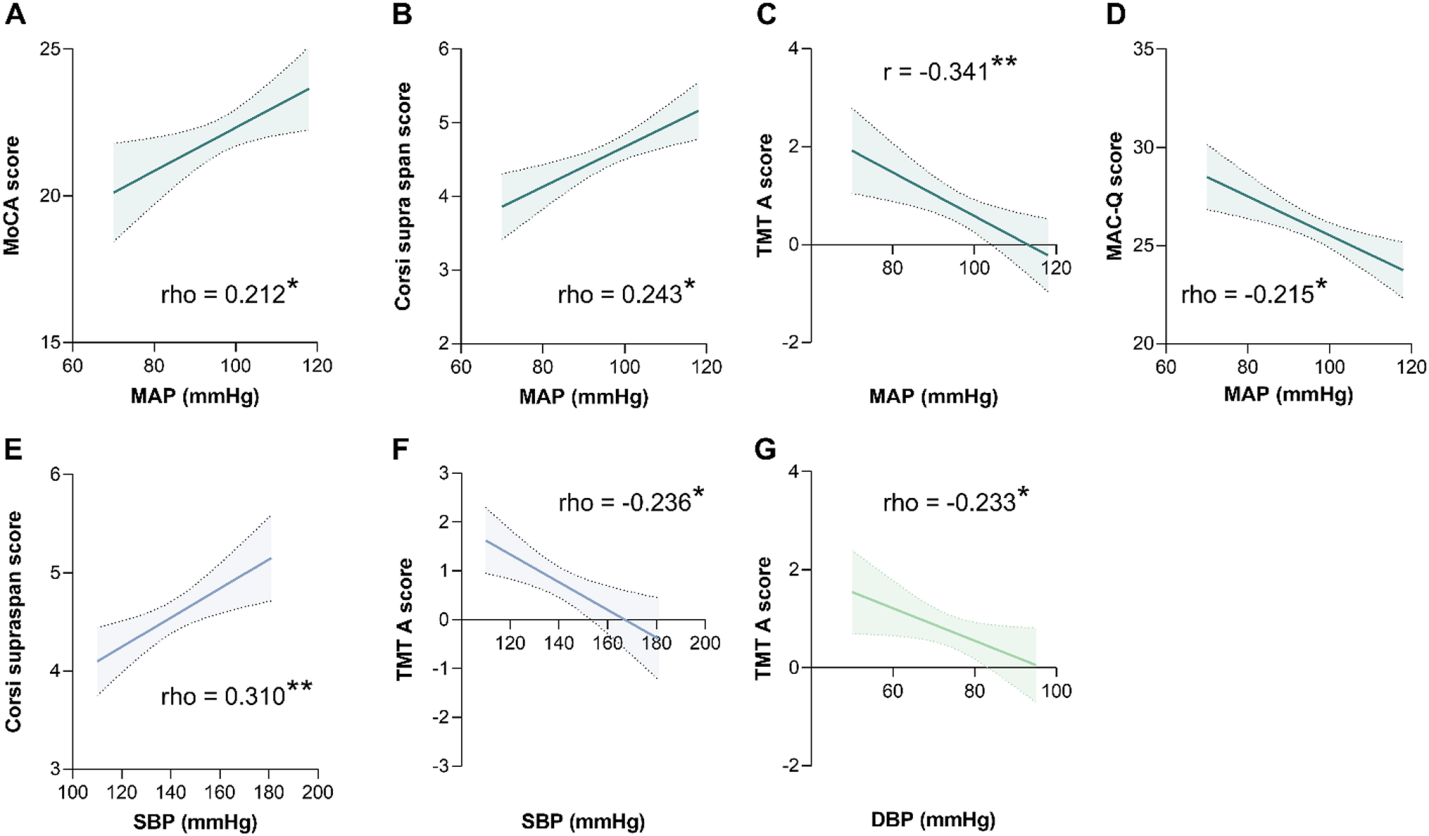
Mean arterial pressure (MAP) exhibits significant correlations with a greater number of cognitive/neuropsychological domains compared to systolic and diastolic blood pressures (SBP and DBP). MAP shows positive correlations with scores on the Montreal Cognitive Assessment (MoCA) (**A**) and Corsi Supra-Span test (**B**) and negative correlations with scores on the trail making test A (TMT A) (**C**) and Memory Complaint Questionnaire (MAC-Q) (**D**). These correlations indicate that patients with lower MAP tend to have poorer cognitive/neuropsychological performances. SBP correlates positively with scores on the Corsi Supra-Span test (**E**) and negatively with scores on TMT A (**F**), while DBP correlates positively with scores on TMT A (**G**). Therefore, SBP and DBP also indicate that lower values are associated with poorer performances, albeit in a smaller number of functional domains. *p < 0.05; **p < 0.01

Figure 1 reports the significant correlations between BP values and metacognitive/cognitive profile. Regarding MAP, a lower value was associated with worse scores on the MoCA (Fig. 1A), Corsi Supra-Span test (Fig. 1B), TMT A (Fig. 1C), and MAC-Q (Fig. 1D). In the case of SBP, a lower value was associated with worse scores on the Corsi Supra-Span test (Fig. 1E) and TMT A (Fig. 1F). In the case of DBP, a lower value was associated with worse scores on the TMT A (Fig. 1G). No significant correlations were found with PP. These findings suggest that MAP might outperform SBP and DBP as haemodynamic parameter directly associated with a better neuropsychological status in the MCI cohort.

By employing the Bonferroni’s correction for multiple comparisons to reduce the risk of type I error, only the correlations encompassing MAP and the Corsi Supra-Span test, MAP and TMT A, as well as SBP and the Corsi Supra-Span test, retained their statistical significance. Despite the utilization of this extremely conservative approach [15], the heightened ability of MAP to correlate with cognitive performance, as compared to SBP and even more so to DBP, is restated.

**Fig. 2:**
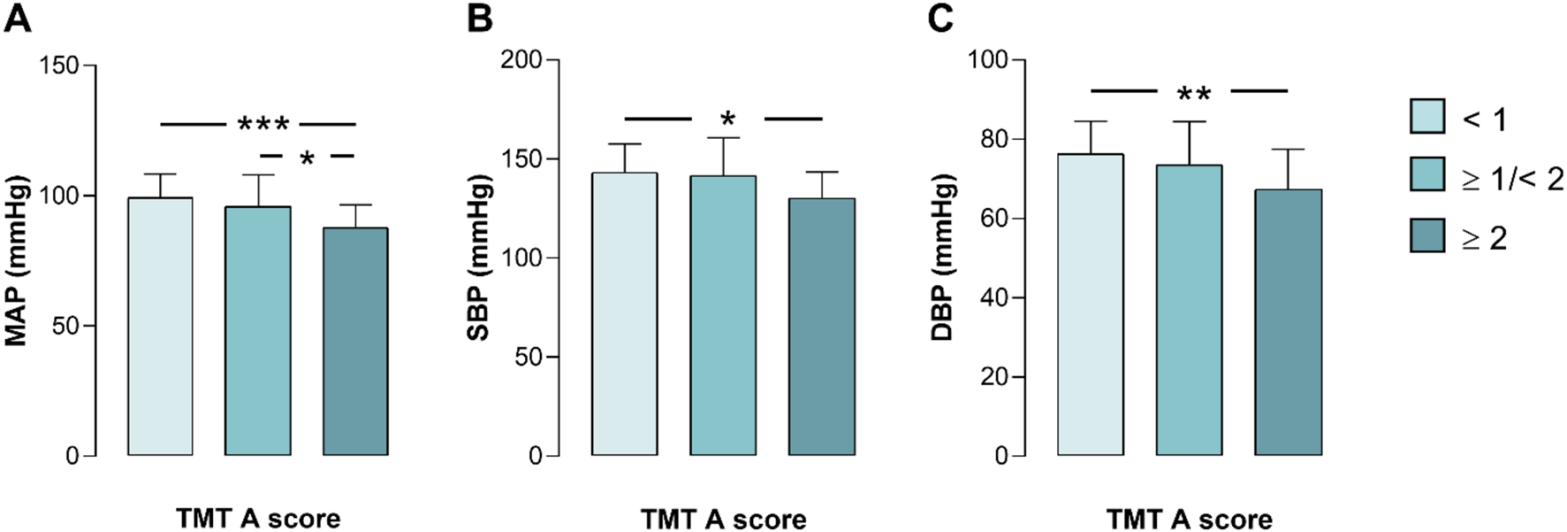
Mean arterial pressure (MAP) better reflects performance in the TMT A test than systolic and diastolic blood pressures (SBP and DBP). MAP values are significantly higher in non-impaired (Z score < 1) and borderline (Z score ≥ 1/ < 2) subjects compared to impaired (Z score ≥ 2) patients (**A**). On the other hand, SBP (**B**) and DBP (**C**) are significantly higher only in non-impaired than impaired subjects. These findings suggest that *MAP* may be a more robust indicator of performance in the *TMT A* test, while *SBP* and *DBP* may have a more limited association with cognitive impairment in this context. *p < 0.05; **p < 0.01; ***p < 0.001

Focusing on the only test initially correlated to all three BP values (i.e., TMT A test), we subdivided the cohort based on the cut-off Z scores. Patients with a score < 1 (n = 49) and ≥ 1/ < 2 (n = 28) had a significantly higher MAP value than patients with a score ≥ 2 (n = 15) (Fig. 2A), while SBP and DBP values were significantly higher only in patients with a score < 1 in comparison to patients with a score ≥ 2 (Fig. 2B and C). Thus MAP, but not SBP or DBP, “discriminates” both non-paired (< 1) and borderline (≥ 1/ < 2) subjects from impaired (≥ 2) ones.

The binary logistic regressions evidenced that no other variables had a significant relationship with the cognitive/neuropsychological domains (Supplemental Table 1). Therefore, BP values affect the functional domains per se.

## Discussion

The present study provides evidence that lower values of SBP, DBP, and MAP correlate with poorer cognitive performances in MCI older adults, while PP had no significant influence.

Our results are in line with previous longitudinal studies that have demonstrated an increased risk of developing dementia in older individuals with persistently low BP values [16, 17]. However, a recent meta-analysis seems to doubt these results, as showed that in older subjects both high SBP and low DBP values were significantly associated with an increased risk of cognitive disorders [3]. Nonetheless, the authors themselves pointed out the need for further investigation into the relationship between cognitive decline and elevated SBP, as other factors such as the APOE allele may potentially modify this evidence. The same meta-analysis confirmed that no association exists between PP and the risk of developing cognitive impairment.

Several mechanisms have been proposed to explain the cognitive decline occurring in older adults with low BP values, including the harmful effects of cerebral hypoperfusion that could lead to oxidative stress, synaptic dysfunction, tau hyperphosphorylation, beta amyloid accumulation, and neuroinflammation [18–20] or the possibility of a “reverse causation” scenario, whereby the onset of dementia itself may impact the central control of BP [21]. Furthermore, it is worth considering that lower MAP values may reflect a compromised capability of cerebral flow autoregulation, which has already been observed in elderly people with MCI [22].

So, what is new in our study? The fact that it addressed a gap in the existing literature. Currently, there are limited or insufficient information available regarding the role of MAP in the context of MCI. Here, we provide valuable insights into the impact of MAP on cognitive health in this population. Remarkably, MAP demonstrated superior capability in reflecting impairment of attention and visuospatial abilities compared to SBP and DBP. Indeed, in addition to being associated with worse scores on a greater number of neuropsychological tests, MAP also exhibited a stronger negative correlation with the TMT A, highlighting its potential for effectively discriminating among impaired, borderline, and non-impaired patients. Besides, MAP, which incorporates both SBP and DBP values, captures changes in both cardiac output and systemic vascular resistance [8]. This comprehensive representation of physiological factors could elucidate its stronger correlation with cognitive decline compared to SBP and DBP alone. The fact that MAP reduction affected above all the results of TMT A, among the neuropsychological tests administered, is particularly interesting. In patients with Alzheimer’s disease, an early reduction in cerebral perfusion occurs in brain areas implicated in the regulation of spatial attention such as the posterior cingulate cortex [23]. Hence, the early impairment of the TMT A in old MCI patients with lower MAP could reflect the initial reduction of these brain areas’ perfusion, which could precede the onset of dementia.

Another interesting outcome of our study was the correlation observed between MAP and TMT A, as opposed to other tests assessing the domain of attentiveness. We explained this finding by considering that TMT A might more effectively capture the decline in psychomotor speed, a phenomenon previously observed in elderly individuals with hypotension, including those afflicted by MCI [24, 25]. Moreover, TMT A evaluates executive functions, a domain hypothesized to be influenced by BP values [26] potentially stemming from modifications in blood flow to the frontal and prefrontal regions of the brain [27]. It is plausible that alternative attention assessment tools could be less adept at quantifying the impairment in psychomotor speed and executive functions among MCI patients. Notably, the performance in TMT B necessitates cognitive shifting capacity, while attention matrices predominantly yield insights into visual selective attention/inhibition and working memory.

An additional noteworthy aspect is that we did not observe any significant influence of commonly used medications for hypertension and cardiac dysfunction on cognitive performance. Previous studies have suggested that RAS-acting agents may reduce the risk of cognitive decline by favourably modulating the renin-angiotensin system, which involves mitigating inflammation, vascular dysfunction, and insulin resistance [28, 29]. However, our result aligns with the current perspective in the medical literature, which does not designate any specific class of antihypertensive drugs as preferable for the prevention of cognitive decline, given the lack of definitive evidence [30].

In summary, our findings lend support to the notion that in older individuals with MCI, excessively low BP values, particularly MAP, are linked to poorer neuropsychological performance, particularly in domains related to attention and visuospatial skills. Therefore, the routine assessment of MAP, alongside traditional SBP and DBP measurements, could be adopted for older patients with MCI. The calculation of MAP can serve as a convenient “surrogate index” of cerebral perfusion, aiding clinicians in therapeutic decision-making (e.g., adjusting antihypertensive treatment) and facilitating diagnostic processes (e.g., ensuring closer neuropsychological monitoring for individuals with lower MAP).

It is important to acknowledge the limitations of our study, including its single-centre design, the use of cross-sectional analysis, the absence of information regarding participants’ genetic profiles and brain imaging tests. This may restrict the generalizability of our findings and hinder the determination of causality. Furthermore, due to the extremely low number of individuals with type 2 diabetes in our cohort, we were unable to adequately examine the potential relationships between cognitive performance and this condition. Lastly, despite our calculation of PP, which can serve as an indicator of large artery stiffness, our study was devoid of a measurement pertaining to carotid stiffness—a factor known to influence cerebral perfusion regulation [31]. Consequently, we were unable to comprehensively estimate its impact on cognitive deterioration within our patient cohort.

However, our research also has merits. Notably, it is the first study to investigate the associations between various BP parameters, including MAP, and cognitive decline in elderly individuals with MCI using a comprehensive cognitive/neuropsychological test battery.

Future large prospective studies might aim to investigate whether an excessive reduction in MAP in older patients with MCI may not only lead to a decline in specific cognitive functions, as observed in our study, but also contribute to an increased conversion rate from MCI to dementia. Additionally, forthcoming research in older adults with MCI might focus on identifying potential BP thresholds below which a reduction in MAP may not be clinically advisable due to the risk of exacerbating cognitive decline.

### Supplementary Information

Below is the link to the electronic supplementary material.

Supplementary file1 (DOCX 19 KB)

## Data Availability

The datasets generated and/or analysed during the current study are available from the corresponding author on reasonable request.
