# Supplementary material for: Association between low values of mean arterial pressure and impaired cognitive performance in older patients with mild cognitive impairment: cross-sectional preliminary findings from the STRENGTH Project
Source: Aging Clin Exp Res. 2024 Jan 28;36(1):9. doi: 10.1007/s40520-023-02668-5 (PMC10822812; doi:10.1007/s40520-023-02668-5)
Supplement: Supplementary file 1 — Supplementary file1 (DOCX 19 KB) [file 40520_2023_2668_MOESM1_ESM.docx]

**Supplemental Table 1.** Influence of blood pressure values, lifestyle, comorbidities, and drugs on TMT A

| **Lifestyle** |  | **Comorbidities** |  | **Drugs** |  |
| --- | --- | --- | --- | --- | --- |
|  | **OR (95% CI)** |  | **OR (95% CI)** |  | **OR (95% CI)** |
|  |  |  |  |  |  |
| **MAP** | 0.94 (0.90-0.99) | **MAP** | 0.94 (0.90-0.99) | **MAP** | 0.95 (0.91-0.99) |
| PASE | 1.00 (0.99-1.01) | hypertension | 0.83 (0.33-2.09) | beta-blockers | 0.64 (0.22-1.88) |
| BMI | 1.06 (0.92-1.22) | dyslipidemia | 0.46 (0.16-1.35) | RAS-acting agents* | 1.00 (0.40-2.50) |
| smoker° | 0.46 (0.10-2.04) |  |  | non-RAS-acting agents^#^ | 1.09 (0.39-3.08) |
| former smoker° | 0.48 (0.19-1.24) |  |  | diuretics^ | 0.94 (0.29-3.01) |
|  |  |  |  |  |  |
| **SBP** | 0.97 (0.94-0.99) | **SBP** | 0.97 (0.95-0.99) | **SBP** | 0.97 (0.95-0.99) |
| PASE | 1.00 (0.99-1.01) | hypertension | 0.90 (0.36-2.30) | beta-blockers | 0.78 (0.27-2.30) |
| BMI | 1.04 (0.91-1.19) | dyslipidemia | 0.51 (0.18-1.47) | RAS-acting agents* | 1.08 (0.43-2.71) |
| smoker° | 0.40 (0.09-1.77) |  |  | non-RAS-acting agents^#^ | 1.07 (0.38-3.01) |
| former smoker° | 0.47 (0.18-1.19) |  |  | diuretics^ | 0.93 (0.29-2.96) |
|  |  |  |  |  |  |
| **DBP** | 0.96 (0.91-0.99) | **DBP** | 0.95 (0.91-0.99) | **DBP** | 0.95 (0.91-0.99) |
| PASE | 1.00 (0.99-1.01) | hypertension | 0.70 (0.28-1.73) | beta-blockers | 0.56 (0.19-1.63) |
| BMI | 1.05 (0.91-1.21) | dyslipidemia | 0.49 (0.17-1.42) | RAS-acting agents* | 0.90 (0.36-2.22) |
| smoker° | 0.46 (0.11-2.03) |  |  | non-RAS-acting agents^#^ | 1.01 (0.36-2.80) |
| former smoker° | 0.51 (0.20-1.28) |  |  | diuretics^ | 0.98 (0.31-3.10) |

MAP, mean arterial pressure; SBP, systolic blood pressure; DBP, diastolic blood pressure; PASE: Physical Activity Scale for the Elderly; BMI: Body Mass Index; RAS: renin-angiotensin system; °*vs* never smoked; *Angiotensin-Converting Enzyme inhibitors, Angiotensin II Receptors Antagonists; ^#^Calcium Channel Blockers, Alpha 2-Adrenoceptors Antagonists; ^Loop Diuretics, Thiazides, Potassium-Sparing Diuretics. Sex was not included because no significant differences for TMT A performances were found comparing women and men.
